# Potential negative impacts and low effectiveness in the use of African annual killifish in the biocontrol of aquatic mosquito larvae in temporary water bodies

**DOI:** 10.1186/1756-3305-3-89

**Published:** 2010-09-16

**Authors:** Martin Reichard, Brian R Watters, Rudolf H Wildekamp, Rainer Sonnenberg, Béla Nagy, Matej Polačik, Stefano Valdesalici, Alessandro Cellerino, Barry J Cooper, Holger Hengstler, John Rosenstock, Ian Sainthouse

**Affiliations:** 1 Institute of Vertebrate Biology, Academy of Sciences of the Czech Republic, Květná 8, 603 65 Brno, Czech Republic; 2 Department of Geology, University of Regina, Regina, Saskatchewan S4 S 0A2, Canada; 3 Musée Royal de l'Afrique Centrale, 3080 Tervuren, Belgium; 4 Zoologisches Forschungsmuseum Alexander Koenig, Adenauerallee 160, D-53113 Bonn, Germany; 5 30, rue du Mont Ussy, 77300 Fontainebleau, France; 6 Institute of Vertebrate Biology, Academy of Sciences of the Czech Republic, Květná 8, 603 65 Brno, Czech Republic; 7 Via Ca' Bertacchi 5, 42030 Viano (RE), Italy; 8 Biology of Aging, Leibniz Institute for Age Research - Fritz Lipmann Institute, Beutenbergstr. 11, 07745 Jena, Germany & Scuola Normale Superiore, Plazza dei Cavalieri 6, 56100 Pisa, Italy; 9 Professor Emeritus, College of Veterinary Medicine, Cornell University, Ithaca, NY 14853, USA; 10 Tegernseerlandstraße 42, 81541 Munich, Germany; 11 Scandinavian Killifish Association, Copenhagen, Denmark; 12 22 Horton Road, Slapton, Leighton Buzzard, Beds., LU7 9DB, UK

## Abstract

Commentary and discussion on a recent paper promoting the use of *Nothobranchius guentheri*, a small African annual fish from the Island of Zanzibar as a tool to control mosquito larvae in temporary bodies of freshwater throughout Africa is presented.

Arguments on major points; (1) expected low success of annual fish introductions, (2) low success of mosquito control in the field, (3) ecological threats, and (4) ethical issues are detailed.

Despite serious problems with mosquito-borne diseases in tropical Africa and elsewhere, we encourage responsible means of biological control of parasite vectors. We show that effectiveness of *Nothobranchius *translocations is low (the previous attempts failed), likelihood of effective mosquito larvae control under field condition is negligible and ecological threats from *Nothobranchius *translocations from within and outside the naturally occurring range are serious. We advocate against the proposed next step of the project, i.e. field trials in Tanzania.

## Letter

Dear Editor

In a recent issue of Parasites & Vectors, Matias and Adrias [1] report on their experiments on food preference of *Nothobranchius guentheri*, a small annual fish from Zanzibar Island (Tanzania), and conclude that the fish may become an ideal "tool to be employed in the eradication of diseases carried by mosquitoes through vector control, particularly in temporary bodies of freshwater". The authors describe previous suggestions to use *Nothobranchius *spp. for mosquito control [2-6] and expand on them to promote *Nothobranchius guentheri *as an ideal species to decrease mosquito larval densities in affected tropical areas. Matias and Adrias used semi-natural experiments at the Poseidon Sciences field laboratory in the Philippines to show that *N. guentheri *preferred to prey upon *Culex quinquefasciatus *larvae over chironomid larvae (*Chironomus plumosus*) and rotifers (*Brachionus *sp.). The authors showed that the presence of three fish per m^2 ^of water surface was sufficient for the total eradication of *C. quinquefasciatus *larvae within four days. Finally, the authors suggested that *N. guentheri *can be conveniently transported in the form of diapausing embryos and then stocked in temporary water bodies that represent habitats for mosquito larvae, and conclude that they can effectively reduce mosquito larval populations [1].

We are well aware of the high risk of malaria and other mosquito-borne diseases in the tropics and we applaud any attempt to reduce the risk of contracting vector-borne diseases in those areas and elsewhere. At the same time, we think that the suggested approach has several important shortcomings, both from practical and ethical points of view. Our opinion is that the suggested practice would be largely ineffective and pose a significant threat to natural ecosystems. Our three major points (expected low success of annual fish introductions; low success of mosquito control in the field; and ecological threats) are detailed below. Some of us (RHW, BRW, BN) were in contact with the lead author of the study before its publication [1], commented on some aspects, and specifically mentioned some of the implications discussed in this response. Some of our concerns were addressed, but many were ignored. We question the overall practicality of the use of annual fishes as mosquito control in temporary water bodies and especially the introduction of *N. guentheri *or any other species of the genus to non-native areas inhabited by indigenous species of the same genus. We believe that the authors of the study should seriously consider modifications to their approach if the decision is made to proceed to the second step of their project, field trials in Tanzania [7].

### Low effectiveness of Nothobranchius fish introductions

*Nothobranchius *fishes require very special habitat conditions. They cannot survive through more than one generation in pools that do not have the necessary habitat conditions, primarily an appropriate substrate for egg survival and development. Perhaps the most comprehensive account on habitat requirements of *Nothobranchius *is by Watters [8] who, based on field observations, experimental data and published studies, discusses the conditions that prevail in *Nothobranchius *habitats and the factors that control their distribution.

*Nothobranchius *fishes have been present in East Africa for many million years and during that time, geomorphological evolution of the landscape, with accompanying changes in drainage patterns, have been very effective in spreading *Nothobranchius *species to all sites that have the conditions necessary for their survival over the long term [8,9]. In other words, if *Nothobranchius *are capable of existing as a viable population in any particular seasonal pool then natural processes have ensured that they are already there. Brian Watters examined more than a thousand *Nothobranchius *habitats [10,11], as well as pools that do not host *Nothobranchius *fishes. In all cases, the reasons for the presence or absence of *Nothobranchius *were primarily determined by the nature of the substrate. Without the particular type of substrate (alluvial vertisols) no eggs can survive the dry season.

During the course of an extensive project in the Kruger National Park (KNP) in South Africa, numerous sites in the northern part of the park, to which *Nothobranchius *had been translocated, were examined [10,11]. Two species of *Nothobranchius *(*N. rachovii*, *N. orthonotus*) occur naturally at only two localities in the park and, for conservation purposes, a decision was made in the mid-1970 s to translocate specimens of these two species to various seasonal pools in a different part of the park. These efforts were carried out during the period 1975-1985 and involved 10 different sites and 15 translocation events. The translocations involved large numbers of fish that were transported at considerable expense (by helicopter in many cases). None of these translocations were successful over even a relatively short term and only in a few cases did the fish survive through a full seasonal cycle. The investigations showed conclusively that the failure of these translocations was due primarily to an unsuitable substrate at the translocation sites that could not sustain the viability of eggs deposited therein by the fish. The only reason that in a few rare cases the translocated populations were able to survive through a seasonal cycle was because the translocations occurred during a particularly wet period. When normal conditions returned the populations became extinct [10].

Further, indirect evidence for the low success of *Nothobranchius *translocation comes from the original translocations by Vanderplank [12] who initiated introductions of *N. taeniopygus *from the vicinity of Old Shinyanga to other regions in Tanzania (Dodoma area), Uganda, Kenya and Swaziland. Numerous sampling efforts have been made in many of these areas over decades since these events, but no evidence for the survival of the introduced *N. taeniopygus *has ever been found [13].

The most important factor that makes the translocation of *Nothobranchius *fishes to new habitats pointless is that any seasonal pool within the overall range of distribution of *Nothobranchius *that is capable of sustaining a population of these fishes will already host one or more species of *Nothobranchius*. It makes no sense to introduce a new, non-native species (or population) into a pool that already hosts other species/populations of the same genus. Research carried out on the factors that control the distribution of *Nothobranchius *species over time [9,14] provides evidence for this as well as the vast field observational experience of the authors who regularly conduct field studies of *Nothobranchius *fishes in the wild.

Tanzania, a potential target of suggested introductions [7], is rich in *Nothobranchius *fishes and they are present in almost every temporary body of water with suitable environmental conditions [15,16]. Unfortunately, it appears that Matias and Adrias have not made a serious effort to research this and to determine what the natural distribution and population density of these fishes are before suggesting a scheme that will have no greater effect on the mosquito population than existing populations of *Nothobranchius *already have.

In summary, introductions to sites presently lacking *Nothobranchius *fishes will very likely have the same negative results (i.e. no population establishment in new habitats) as previous attempts.

### The effectiveness of eradication of mosquito larvae in the wild

Most *Nothobranchius *species readily feed on mosquito larvae, including larval *Culex *spp. [15] and mosquito larvae are used as the most common diet for captive *Nothobranchius *[16]. This finding has been quantitatively confirmed by Matias and Adrias [1]. Studies on the diet of *Nothobranchius *in the wild are rare and Matias and Adrias cite a work which found undetermined mosquito larvae as main food item for a *Nothobranchius *species from Somalia. We suppose that the exact reference in the manuscript is incorrect as it suggests that the statement refers to findings from an experimental study concerning the resistance of egg chorion to external chemical damage [17], and we deduce that this information comes from a WHO (World Health Organization) report by Wildekamp [4] on *N. microlepis *or previous WHO assignment reports [18,19] describing the diet of *Nothobranchius patrizii *(then determined as *N. palmqvisti*). However, Matias and Adrias selectively used only a part of the study that supports their results. The report by Wildekamp [4] indeed describes the frequent presence of mosquito larvae in *N. microlepis *stomachs. This report also contains data showing that *N. microlepis *had a clear preference for small planktonic crustaceans and their nauplii. The consumed mosquito larvae, mentioned in the report [4] were taken during experimental trials when mosquito larvae were the only food offered. *Nothobranchius microlepis *may be ecologically distinct from *N. guentheri *used by Matias and Adrias since it was discovered later that *N. microlepis *have specially adapted gill rakers to prey on small food items [20]. Mosquito larvae were frequently found in the diet of *N. cyaneus *(= *N. jubbi*) but they also frequently preyed on larger sized planktonic crustaceans and *Coryxa *nymphs (Hemiptera), and the conclusion that *Nothobranchius *have a preference for mosquito larvae is certainly not supported by [4] despite showing that mosquito larvae were frequently consumed (together with some other prey types) by some species.

Another recent study examined the diet of three sympatric species of *Nothobranchius *in southern Mozambique and found that mosquito larvae were extremely rare items in the diet of all three *Nothobranchius *species studied [21], while crustaceans (in *N. furzeri *and *N. rachovii*, two species with body size and morphology comparable to *N. guentheri*) and coarse insect larvae such as Odonata, Ephemeroptera and Coleoptera (in *N. orthonotus*, a larger species) were the primary prey. We acknowledge the possibility that mosquito larvae may have already been eradicated if they constituted the preferred food and hence were not encountered during diet analysis, but the conservative interpretation is that mosquito larvae may not be as important component of the *Nothobranchius *diet in the wild, especially considering that the natural habitats of annual fishes and mosquito larvae are not identical.

Yet another pitfall with regard to the ability of *Nothobranchius *to eradicate mosquito larvae in the wild is that very substantial numbers of larvae would survive in the wide margins of natural pools overgrown by thick grass vegetation [14] in water too shallow for *Nothobranchius *fishes to reach. Mosquito larvae tend to be concentrated in the marginal zones of water bodies [22] and anopheline larvae typically reside among vegetation with limited motion and hence do not attract visual predators (such as *Nothobranchius*) [23]. This sedentary behaviour is in contrast to active swimming by culicine larvae used in the experiments by Matias and Adrias [1]. Even if *Nothobranchius *fishes were able to eradicate mosquito larvae in temporary habitats in which they are capable of surviving, their special habitat requirements prevent them from inhabiting many typical habitats of mosquito larvae, such as almost any type of receptacle capable of holding even very small amounts of rainwater (including waste items such as bottles, cans and any water containers in households) or cattle hoofprints (very typical feature of African countryside with a human settlement). These small bodies of water are significant habitats for mosquito larvae, but are impossible to stock with predators because of their abundance and small size [24]. This points to the serious logistical impracticality of the proposed scheme.

### Ecological threats from introductions

Countries of sub-Saharan Africa are richly endowed with many species of *Nothobranchius *and related genera of annual fishes. For example, the coastal region of Tanzania alone includes more than 20 species of *Nothobranchius*. The assertion that *N. guentheri *is native to Tanzania [1] is correct but very imprecise, since that species is endemic to Zanzibar Island and does not occur on the African mainland [15]. As pointed out by Matias and Adrias [1], there are indeed localities with more than a single *Nothobranchius *species [4,14,15], but this does not diminish the potential threat of hybridization between indigenous and introduced species. There are numerous accounts of introgressive hybridization between native and introduced congeneric species in many organisms [25], including killifishes [26]. For example, hybridization between a non-native species of pupfish (*Cyprinodon variegatus*) and its native congener not only resulted in extinction of the native species via hybridization but also facilitated a further expansion of non-native pupfish and its hybrid swarm [27]. Within the genus *Nothobranchius *alone, Reichard and Polačik [28] experimentally showed that there were almost no reproductive isolating barriers between an island and mainland populations of *N. korthausae *despite their significant morphological and genetic differentiation leading some authors to assign the mainland populations as a separate species [29]. The risk of hybridization is especially high in taxa where sexual selection plays an important role in reproductive success [30] as is the case in *Nothobranchius *with brightly coloured males [31]. Native species (or populations) may show a preference for partners from non-native populations/species [32,33], which promotes rapid extinction via extensive hybridization [34].

The second potential threat to native *Nothobranchius *species arises from interspecific competition [25]. The ecological niche of most *Nothobranchius *fishes is similar [15,16,21] and, at present, there is serious lack of information on how *Nothobranchius *communities (presence/absence of species and their relative abundance) are shaped. Despite the fact that seemingly ecologically similar species may co-occur syntopically [14], in most habitats with multiple sympatric *Nothobranchius *species, the species present do appear to be ecologically separated [15].

Finally, there is an important risk of introducing diseases [35]. Introduced fish may transmit diseases contracted during captive breeding into the natural environment, with devastating consequences on natural populations [36]. Infections of serious pathogens such as the microporidian *Glugea *sp. are common in *Nothobranchius *cultures [37].

In summary, we believe that transport of *Nothobranchius *fish and eggs outside their current range is irresponsible and may have significant effects on the distribution of native congeners as well as other organisms in aquatic communities.

### Ethical issues and incorrect statements

We believe that the Matias and Adrias initiative [1] is driven by the necessity to develop a new approach to combat infectious insect-borne diseases. The authors, both affiliated with the Poseidon Science Foundation, a non-profit branch of Poseidon Sciences biotechnological company with a large portfolio of insect (mosquito) control products [38], stated that there were no competing interests and hence we assume that the study constitutes an attempt to generously contribute to a mosquito control programme without any prospect of commercial or other interests. We welcome this attempt, but urge the authors to adhere to the mission targets of the Poseidon Sciences Foundation that include protection and preservation of the aquatic environment as their first aim [7].

We think that the recommendations given in the paper [1] pose a potentially very significant threat to natural communities in temporary freshwater pools across the African continent and adjacent islands. We are pleased that authors are aware that the introduction of *N. guentheri *to non-native areas within Tanzania and elsewhere, is inappropriate. A solution offered by Matis and Adrias is that another species with wider distribution, such as *N. melanospilus*, may be used instead. While this may represent a step forward from the original plans, it should be pointed out that: (1) *N. melanospilus *populations are variable and have a strong phylogeographical pattern, which is common to all *Nothobranchius *species studied to date [39], and hence even translocation within its wide range is not recommended; (2) *N. melanospilus *will probably hybridize with related and currently allopatric/parapatric species such as *N. makondorum *or *N. lucius*; (3) *N. melanospilus *may pose a risk to other native *Nothobranchius *via competition (including its indirect effects [25]); (4) experimental data obtained for *N. guentheri *may not be transferable to other species of the genus [40].

The study by Matias and Adrias misquotes the results of previous studies (see an example above on mosquito larvae as the dominant natural prey) and includes some incorrect statements. For example, the statement that juvenile *Nothobranchius *do not feed for the first three days is highly inaccurate since the juveniles must start feeding within a few hours after hatching (certainly within less than a day) in order to survive [16]. Another important fact is that the photograph published in the Matias and Adrias paper as *N. guentheri*, the study species, actually depicts a different *Nothobranchius *species (an undescribed *N*. sp. aff. *kirki *from Malawi), that is morphologically very different to *N. guentheri *and likely not even closely related. It is therefore unclear whether *N*. sp. aff. *kirki *or *N. guentheri *were used for the experiments. Also a map depicting the distribution of African annual fishes (Figure 8 in [1]) is largely incorrect, even if other African genera with potentially annual species (*Fundulopanchax*, *Callopanchax*, and *Raddaella*) are included.

In conclusion we reiterate that we are aware of serious problems with mosquito-borne diseases in tropical Africa and elsewhere, and we encourage responsible means of biological control of parasite vectors. There are several cases where native fish species were found to be effective predators of mosquito larvae [e.g. [41,42]] but there are also many cases where the ecological balance has been catastrophically affected [e.g. [25,43,44]]. We believe that any serious attempt to advocate particular fish species as a biocontrol agent needs to be based on sound scientific attempts to assess both the ability of species to control the parasite vector or pest, and the potential impact on natural ecosystems [23]. Otherwise, there is a significant risk of repeating mistakes made in the past decades, such as the introduction of *Gambusia holbrooki *into many countries with catastrophic consequences for native species [43,44] and with no effect on mosquito populations [45,46].

## Competing interests

The authors declare no financial, academic or intellectual competing interests. We acknowledge that our opinions are based on our personal views on how the relationship between environmental protection and development should be assessed.

## Authors' contributions

MR, BRW, RHW initiated the report. MR drafted the manuscript. All authors contributed to and approved the final version of the manuscript.

## Authors' information

The authors include active researchers on African killifish ecology and evolution and specialized *Nothobranchius *fish keepers, members of several national killifish keeping societies. The authors regularly collect *Nothobranchius *fishes in their natural habitats.
